# The Harveian Society

**Published:** 1896-11-28

**Authors:** 


### THE HARVEIAN SOCIETY.

A well-known manual says: "Chronic interstitial nephritis is a disease of middle and advanced life; it occurs with extreme rarity under twenty years of age, and most frequently between forty and sixty." Such being the case, considerable interest attaches to the paper read by Dr. Leonard Guthrie before the Harveian Society on November 19th on "Chronic Insterstitial Nephritis in Childhood," a paper founded on seven cases in which that disease had been observed in children between five and fourteen years of age, and verified by post-mortem examination, and on other cases as yet incomplete. Emphasis was properly laid on the likelihood of this disease being overlooked from its not being suspected. As in adults, dropsy is mostly absent, in some cases entirely so; and although in people of more advanced age the well-known symptoms of the disease lead easily to its recognition even in the absence of dropsy, there is no doubt that, as the author pointed out, kidney disease in children is so very commonly associated with dropsy that the absence of this system is apt to mislead.

The physical signs which attend the disease are progressive emaciation, which may date from some illness in early life often of the nature of gastro-enteritis, anæmia, dryness, absence of perspiration, coarseness and lack of elasticity of the skin (often associated with a good deal of pigmentation), and the well-recognised cardio-vascular changes. Special stress was laid by Dr. Guthrie on the occurrence of pigmentation as a valuable diagnostic sign, and it was suggested that it might be connected with some damage to the adrenals from the acute inflammatory condition, which he believes usually exists in the early stage of this complaint.

The question of the ætiology of interstitial nephritis, as found in these small patients, is one of considerable importance, and we cannot say that we are quite in agreement with Dr. Guthrie in this matter. After discussing various hypotheses, he expressed himself as leaning strongly to the view that it was the last stage of a primary acute interstitial nephritis. When, however, we consider the circumstances in which the same condition arises in adults, and the apparent influence of continuously acting or often repeated causes in its production, such as plumbism or gout, it seems difficult to see why the same condition in the child should be attributed to an antecedent acute disease, which in many cases cannot even be proved to have occurred. We need not point out how differently one must regard the hopefulness and even the utility of treatment, according as we look upon the pathological state as due to a necessary and inevitable contraction of the products of a bygone inflammation, or as a gradual effect of a tissue poison manufactured day by day in consequence of some possibly curable disorder affecting the assimilation of the food or the metabolism of the tissues. The prime importance of the paper lay in drawing attention to a condition which is undoubtedly often overlooked.

